# Supplementary material for: Isolated Radial Nerve Palsy in a Newborn Due to a Congenital Myofibroma: A Rare Case of Peripheral Nerve Injury
Source: Children (Basel). 2024 Sep 15;11(9):1126. doi: 10.3390/children11091126 (PMC11430799; doi:10.3390/children11091126)
Supplement: Supplementary file 1 [file children-11-01126-s001.zip › children-3115235-supplementary.pdf]

CAUSES	RISK FACTOR	DIFFERENTIAL DIAGNOSIS
• Bandl's ring (uterine ring)<br>• Constriction band<br>• Prolonged application of arm cuff for blood pressure measurement<br>• Tumors<br>• Septic arthritis of the shoulder<br>• Infantile cortical hyperostosis (Caffey disease)	• Caesarean section<br>• Gestational diabetes<br>• Prolonged labor<br>• Failure to progress	• Brachial plexus birth palsy<br>• Humerus shaft fracture<br>• Clavicle fractures<br>• Cerebral insult<br>• Arthrogryposis

Table S1. Neonatal upper limb palsy
